# Molecular characterization, clinical value, and cancer–immune interactions of genes related to disulfidptosis and ferroptosis in colorectal cancer

**DOI:** 10.1007/s12672-024-01031-y

**Published:** 2024-05-24

**Authors:** Xianqiang Liu, Dingchang Li, Wenxing Gao, Peng Chen, Hao Liu, Yingjie Zhao, Wen Zhao, Guanglong Dong

**Affiliations:** 1 Medical School of Chinese PLA, Beijing, 100853 China; 2 Department of General Surgery, The First Medical Center, Chinese PLA General Hospital, Beijing, 100853 China; 3 School of Medicine, Nankai University, Tianjin, 300071 China

**Keywords:** Disulfidptosis, Ferroptosis, Tumor microenvironment, Prognosis, Chemotherapy, Immunotherapy

## Abstract

**Background:**

This research strived to construct a new signature utilizing disulfidptosis-related ferroptosis (SRF) genes to anticipate response to immunotherapy, prognosis, and drug sensitivity in individuals with colorectal cancer (CRC).

**Methods:**

The data for RNA sequencing as well as corresponding clinical information of individuals with CRC, were extracted from The Cancer Genome Atlas (TCGA) dataset. SRF were constructed with the help of the random forest (RF), least absolute shrinkage and selection operator (LASSO), and stepwise regression algorithms. To validate the SRF model, we applied it to an external cohort, GSE38832. Prognosis, immunotherapy response, drug sensitivity, molecular functions of genes, and somatic mutations of genes were compared across the high- and low-risk groups (categories). Following this, all statistical analyses were conducted with the aid of the R (version 4.23) software and various packages of the Cytoscape (version 3.8.0) tool.

**Results:**

SRF was developed based on five genes (ATG7, USP7, MMD, PLIN4, and THDC2). Both univariate and multivariate Cox regression analyses established SRF as an independent, prognosis-related risk factor. Individuals from the high-risk category had a more unfavorable prognosis, elevated tumor mutational burden (TMB), and significant immunosuppressive status. Hence, they might have better outcomes post-immunotherapy and might benefit from the administration of pazopanib, lapatinib, and sunitinib.

**Conclusion:**

In conclusion, SRF can act as a new biomarker for prognosis assessment. Moreover, it is also a good predictor of drug sensitivity and immunotherapy response in CRC but should undergo optimization before implementation in clinical settings.

**Supplementary Information:**

The online version contains supplementary material available at 10.1007/s12672-024-01031-y.

## Introduction

Colorectal cancer (CRC) is the third most common gastrointestinal cancer, with the second highest cancer-linked mortality rate globally. Additionally, CRC is the fourth most common disease and the second primary contributor to cancer-related mortality in the US [1, 2]. Currently, surgery is regarded as the optimal therapy option for primary tumors. However, most individuals present with advanced CRC at diagnosis and are lost to surgery. Taking into account the high rates of CRC-related morbidity and mortality, more effective prognostic models need to be developed. Furthermore, valuable biomarkers need to be identified to classify individuals with distinct characteristics into different groups (categories) for predicting the efficacy of immunotherapy. Chemotherapy, radiotherapy, and immunotherapy are among the most frequently applied approaches to treat advanced CRC. Almost 50% of individuals with stage II–III rectal cancer and 66% with stage II–III colon cancer need adjuvant chemotherapy [3]. Additionally, 54% of individuals relapse post-neoadjuvant therapy. Furthermore, immunotherapy is a new therapy option for various tumor forms, and several studies have established its effectiveness in this regard [4–6]. Common immunotherapeutic methods include immune checkpoint inhibitors (ICIs), monoclonal antibodies, and cellular therapies [7]. Anti-PD-1 therapy has been demonstrated as exhibiting favorable responses in various human malignancies, such as glioma, melanoma, and even renal cell carcinoma [8–11].

Literature reports have established that the tumor microenvironment (TME) contributes to the malignant behavior of tumors and also influences the subsequent response to immunotherapy. TME comprises multiple components like blood vessels, tumor cells, infiltrating immune cells, tissue fluid, stromal cells, and cytokines [12, 13]. Apart from classical apoptosis, ferroptosis, necroptosis, and scorch death are all mechanisms for regulated cell death (RCD). Ferroptosis, a regulated form of iron-dependent cell death, is induced by the lethal accumulation of lipid-based reactive oxygen species (ROS) resulting from iron-dependent lipid peroxide buildup. This process primarily relies on iron-mediated oxidative damage and cell membrane damage, particularly when the glutathione (GSH)-dependent repair system for lipid peroxides is compromised [14, 15]. Ferroptosis emerges as a pivotal factor across diverse diseases, including cancer, inflammatory diseases, and more, underscoring its potential as a therapeutic target [15]. Additionally, the relation of ferroptosis with immunotherapy and tumor cell proliferation has been comprehensively explored, and the relevant findings have been implemented in different aspects of cancer treatment [16–18].

Recent research demonstrated that the uptake of cystine mediated by solute carrier family 7 member 11 (SLC7A11) contributes significantly to supporting glutathione biosynthesis and repressing ferroptosis and oxidative stress [18]. SLC7A11 regulates the abnormal accumulation of cystine and other disulfides through different pathways [19–21], thereby resulting in disulfide stress and, ultimately, rapid cell death termed disulfidptosis.

However, SRF has not yet been demonstrated in the survival prognosis, tumor immune microenvironment, TMB, immunity, and clinical treatment of CRC patients. We still lack direct evidence of the predictive power of SRF on CRC prognosis and immunotherapy. The current study analyzed the expression of ferroptosis-related genes (FRGs) linked to CRC and disulfidptosis in a systematic way. It also investigated their influence on prognosis, tumor development, TME, and response to treatment in CRC. The obtained findings can potentially contribute to developing viable immunotherapies for CRC. For this purpose, a comprehensive genomic analysis was conducted by characterizing the molecular correlates of disulfidptosis in CRC and reporting the crosstalk between disulfidptosis and ferroptosis regulators at multiple histological levels. SRF was both developed and authenticated to predict the reaction to immunotherapeutic and chemotherapeutic agents. Overall, this research proposes a scoring model targeting disulfidptosis and ferroptosis regulators to distinguish individuals with CRC who are eligible for immunotherapy. Moreover, it can also be employed to predict sensitivity to chemotherapeutic agents.

## Materials and methods

### Data collection

Transcriptome data, CNV data, data regarding somatic mutations, RNA expression information, and clinical data of individuals with CRC were retrieved from the Cancer Genome Atlas database (https://portal.gdc.cancer.gov/). These individuals were segregated randomly into two cohorts—namely training and validation (ratio 7:3) for the purpose of developing and validating the prediction model, respectively. External validation data for clinical parameters and standardized gene expression correlations were obtained from GSE38832 in the GEO repository. These data were generated using the Affymetrix Human Genome U133 Plus 2.0 Array (GPL570) platform. However, certain samples were not included in subsequent investigations owing to missing clinicopathological or survival data. In addition to this, sets of FRGs and SRGs were obtained from literature studies and the FerrDb (http://www.zhounan.org/ferrdb/current/), respectively [22].

### Consistent clustering analysis of FRGs and SRGs

Consensus clustering is applied to define various SRF correlation patterns with the help of the k-means algorithm. The number and consistency of clusters were analyzed with the aid of a consensus clustering algorithm that is part of the ‘ConsensuClusterPlus’ package [23]. Moreover, gene set variation analysis (GSVA) facilitated the identification of variations between the biological roles of FRGs and SRGs via a KEGG gene set (c2.cp.kegg.v7.2.gmt) [24].

### Correlation of molecular patterns with clinical attributes and CRC prognosis

To ascertain the clinical value of clusters produced via consensus clustering, the correlation of molecular patterns was investigated with the clinical attributes and survival outcomes of individuals with CRC. Clinical variables included sex, age, T-, N-, M-, and TNM-stage. Additionally, Kaplan–Meier (KM) analysis was performed with the help of packages named ‘survminer’ and ‘survival’ to discover variations in Overall survival (OS) across various molecular patterns.

### Identification of differentially expressed genes (DEGs) and functional enrichment analysis

A package called ‘limma’ with |log2-fold change (FC)|≥ 0 and *P*-value < 0.05 facilitated the identification of DEGs across different subgroups of SRF genes. These DEGs were, in turn, applied for KEGG analysis with the help of the “clusterProfiler” package [25].

### Development of a prognostic scoring scheme using SRF genes

The correlation between SRGs and FRGs was examined to identify specific FRGs that were closely associated with SRGs (|r|= 0.3, *P* < 0.05). Moreover, univariate Cox regression analysis facilitated the identification of candidate genes linked to prognosis (|HR|> 0, *P* < 0.05). We performed “least absolute shrinkage and selection operator (LASSO) regression analysis” with tenfold cross-validation using the “glmnet” R package to determine the penalty regularization parameter λ on these twelve genes [26] and using the “random forest (RF)” algorithm, a supervised machine learning approach to rank SRF genes for predicting the patient’s prognosis. We estimated the predictive performance of the risk model using tenfold cross-validation. We identified six genes with significance > 0.3 as feature genes. Variable importance in the random forest models were measured through mean decrease in accuracy and the Gini Index. The RF, LASSO, and stepwise regression algorithms aided the selection of five genes to develop the SRF gene signature.

The risk score was computed by multiplying gene expression with the coefficient of the variable:

$$\begin{aligned} {\text{Risk score}} & = {\text{Expression}}\left( {{\text{mRNA1}}} \right) \times {\text{Coef}}\left( {{\text{mRNA1}}} \right) + {\text{Expression}}\left( {{\text{mRNA2}}} \right) \\ & {\kern 1pt} \quad \times {\text{Coef}}\left( {{\text{mRNA2}}} \right) + ...{\text{Expression}}\left( {{\text{mRNAn}}} \right) \times {\text{Coef}}\left( {{\text{mRNAn}}} \right) \\ \end{aligned}$$

The predictive performance of SRF was assessed through receiver operating characteristic (ROC) curves, time ROC curves, KM analysis, and principal component analysis (PCA). In addition to this, the ‘survival’ package was used for the implementation of the univariate and multivariate Cox regression analyses so as to ascertain the predictive efficiency of risk scores and other clinical indicators. The same formula was utilized to compute the risk scores of all individuals in the TCGA train cohort and TCGA test cohort. Moreover, the efficacy of SRF in prognosis prediction was validated in the external cohort GSE38832.

### Role of SRF genes in predicting immunotherapy response

The packages called ‘GSVA’ and ‘GSEABase’ aided the implementation of the single-sample gene set enrichment analysis (ssGSEA) for the quantification of immune cells and their function for individual samples. The CIBERSORT algorithm was also applied with the help of the package called ‘immunedeconv’ to ascertain the abundance of infiltrating immune cells in the TME of CRC. The expression of common immune checkpoints was compared between the high- and low-risk categories. The Tumor Immune Dysfunction and Exclusion (TIDE) algorithm aided the calculation of TIDE scores to estimate the subsequent reaction to immunotherapy [27]. Following this, TIDE scores were compared across the high- vs. low-risk categories. Furthermore, a real-world immunotherapy cohort was employed to estimate variations in the reaction to anti-PD-1 therapy in high- vs. low-risk individuals with CRC.

### Somatic mutation analysis

Since mutations can influence CRC prognosis, variations in mutations were analyzed in the high- vs. low-risk categories. The 20 leading gene mutations were identified in the high- and low-risk categories, and the obtained results were visualized on waterfall plots with the help of the package called ‘maftool.’ Furthermore, the TMB was computed for each sample and subsequently compared in the high- vs. low-risk categories.

### Construction of a predictive nomogram

Nomograms are effective clinical predictors of risk scores and other clinicopathological attributes, especially 1-, 3-, and 5-year OS in individuals with CRC.

For this study, a curve of calibration was plotted to validate the clinical reliability of the established nomogram. Moreover, to validate the prognostic performance of SRF in CRC, SRF was compared against prognostic models reported in literature studies with the help of the ‘ggDCA’ and ‘rms’ packages.

### Role of SRF genes in anticipating drug sensitivity

The package in R called ‘pRRophetic’ aided the selection of effective drugs from a set of > 300 drugs for individuals in the high- and low-risk categories. Subsequently, the connection between drug sensitivity and risk scores was ascertained, with sensitivity indicators expressed as IC50 values.

### Anticipating miRNA targets and developing miRNA–mRNA networks

The R package ‘limma’ helped to screen for differentially expressed miRNAs in the TCGA cohort, wherein the critical *P*-value was set to 0.1 to avoid misses. Additionally, TargetScan (https://www.targetscan.org/) was used to distinguish the targets of miRNAs. It is an online tool that recognizes miRNA targets using mRNA seed regions by seeking complementary specific sequences. For this, predicted miRNAs of target genes were identified with differential miRNAs taking intersections. Then, these miRNA–mRNA regulatory networks were used along with the Cytoscape tool to visualize the interactions of miRNAs with their potential targets.

### Statistical analysis

R (version 4.1.2) software was used for statistical analyses as well as graph plotting. Risk group, sex, age, and tumor stage were integrated to create a nomogram for anticipating the 1-, 3-, and 5-year OS. Furthermore, these plots were calibrated against the Hosmer–Lemeshow test to ascertain if the predictions were consistent with the actual results of the ‘rms’ package in R. All executed statistical analyses were two-sided, with a *P*-value of < 0.05 indicating the statistical significance level.

## Results

### Genetic and transcriptional modifications in disulfidptosis regulators in CRC

**Fig. 1:**
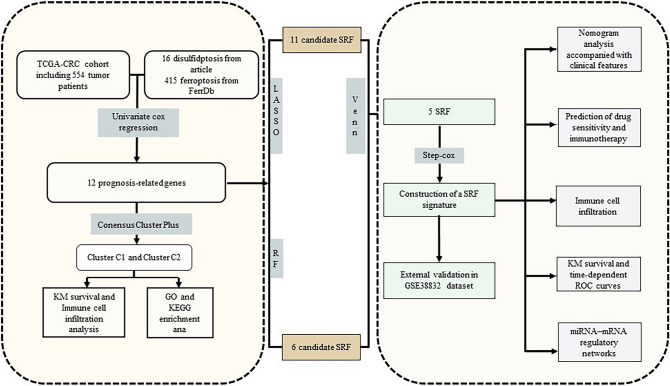
Flow chart of this study

**Fig. 2:**
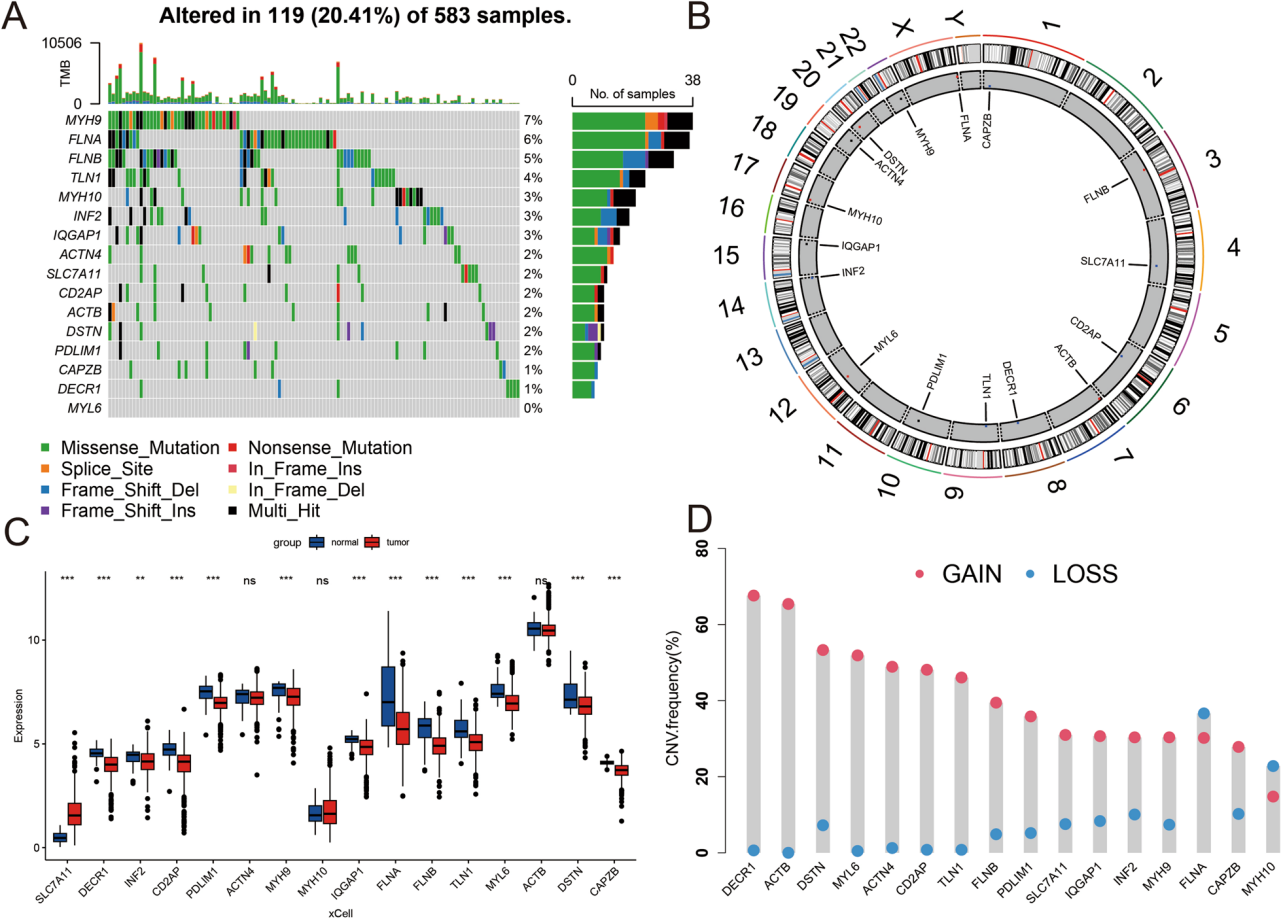
Landscape of genetic mutations and expression of disulfidptosis regulators in CRC. **A** Frequency of disulfidptosis regulator mutations in 583 individuals with CRC from the TCGA cohort. Columns depict individuals. The bar graph above represents TMB. The numbers towards the right depict mutation frequency. The bar graph on the right represents variant type proportion. **B** Location of disulfidptosis regulators on chromosome 23. **C** Bulk sequencing depicting disulfidptosis regulator expression in CRC versus adjacent non-tumor samples in the TCGA-CRC cohort (n = 585). **D** Frequencies of CNV gain, loss, and non-CNV among SRF genes. (*P < 0.05; **P < 0.01; ***P < 0.001)

Illustrates the methodology of this study (Fig. 1A). A total of 16 disulfidptosis regulators were analyzed. To examine genetic alterations in disulfidptosis regulators, the frequency of non-silent somatic mutations was determined in CRC. Of the 583 CRC samples, 115 (11.94%) samples harbored mutations in SRGs. MYH9 (7%), FLNA (6%), and FLNB (5%) had the highest mutation frequency, whereas no mutations were found in MYL6 (Fig. 2A). No variations were observed in patient survival between mutated vs. non-mutated individuals in the TCGA-CRC cohort. (Supplementary Figure S1). The chromosomal location of disulfidptosis regulators is illustrated in Fig. 2B. Furthermore, the mRNA expression of disulfidptosis regulators was compared in CRC tissues versus adjacent non-tumor tissues in the TCGA-CRC cohort, and most genes were found to be lowly expressed in the CRC tissue (Fig. 2C). The frequency of copy number amplification was elevated in the 16 SRGs (Fig. 2D). Moreover, upon examining the connection between genetic variations and mRNA expression, it was ascertained that genes with copy number amplification had low mRNA expression of SRGs. Overall, these results exhibit a high degree of heterogeneity in the genetic landscape and expression of disulfidptosis regulators in CRC versus normal tissue samples, suggesting that an imbalance in disulfidptosis regulator expression contributes significantly to the onset and progression of CRC.

### Identification of SRGs associated with FRGs

**Fig. 3:**
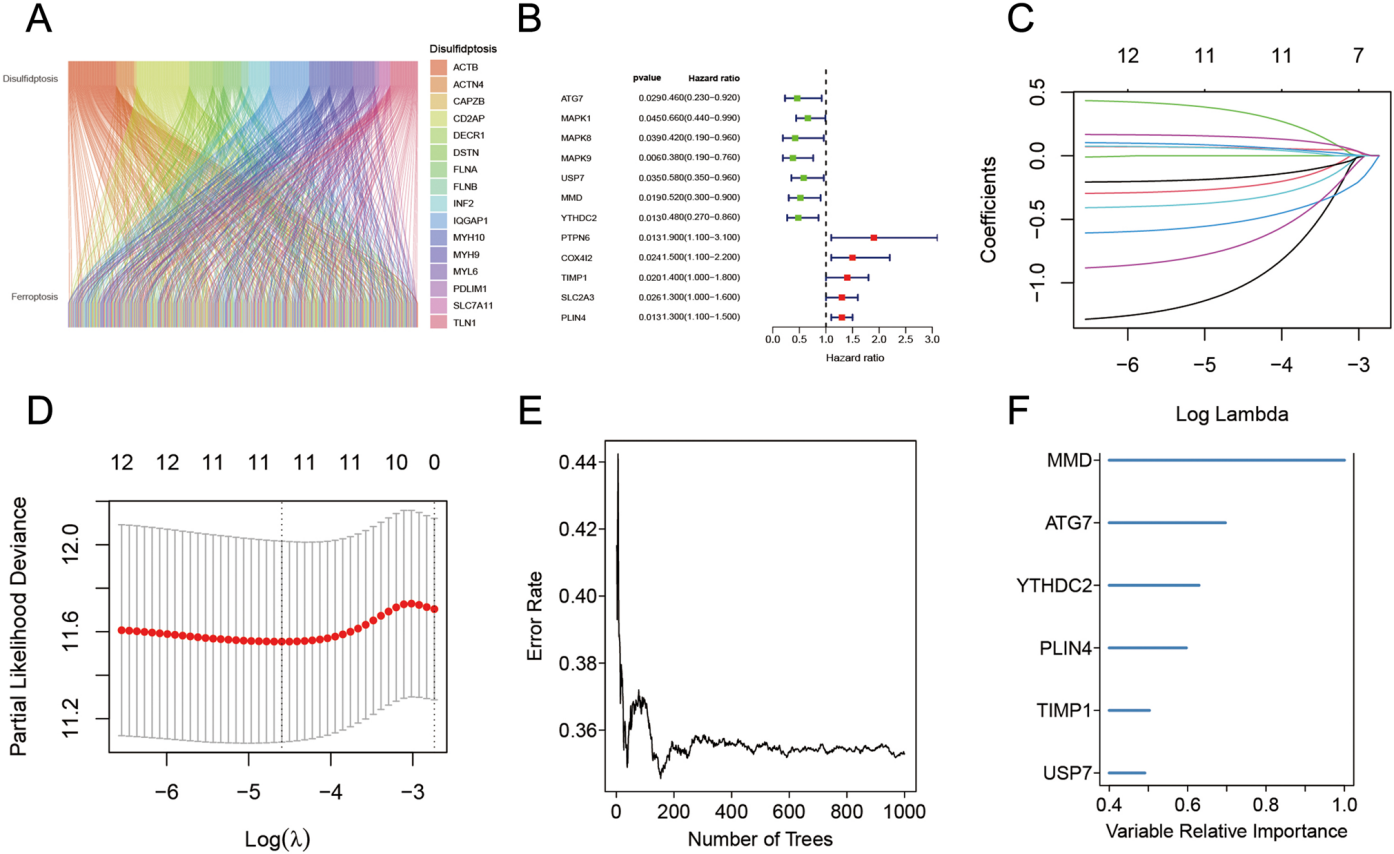
Crosstalk between disulfidptosis and ferroptosis regulators in cancer. **A** Sankey diagram of SRF. **B** Forest plot illustrating the prognosis of SRF regulators for CRC in TCGA. **C** Tenfold cross‐validation for parameter choice in the LASSO model. **D** Profiles of LASSO coefficient. **E** The association of the number of RF trees with error rates. **F** Ranking of the relative significance of genes

To examine the potential relationship between different cell death pathways in the training cohort, crosstalk between disulfidptosis and ferroptosis regulators was analyzed. Furthermore, correlation analysis facilitated the identification of important regulators contributing to the interrelationship (Fig. 3A). A total of 219 FRGs were found to be closely linked to the 16 SRGs. Of these 219 FRGs, 12 genes were reported to be linked to the prognosis of patients with CRC. Figure 3B. Moreover, these candidate genes were inputted into the LASSO (Fig. 3C and D), RF (Fig. 3E and F), and stepwise Cox algorithms. Five genes—namely ATG7, USP7, MMD, PLIN4, and YTHDC2, were eventually selected for the construction of SRF. Risk scores were also computed according to the expression and coefficients of these genes as depicted:

$$\begin{aligned} {\text{Risk score}} = & \left( {{\text{expression level of ATG7}} \times \left[ { - {\text{1}}.{\text{239}}} \right]} \right) + \left( {{\text{expression level of USP7}} \times \left[ { - 0.{\text{8}}} \right]} \right) \\ & + \left( {{\text{expression of MMD}} \times \left[ { - 0.{\text{712}}} \right]} \right) + \left( {{\text{expression level of PLIN4 }} \times \left[ {0.{\text{255}}} \right]} \right) + \left( {{\text{expression level of YTHDC2}} \times \left[ { - 0.{\text{656}}} \right]} \right) \\ \end{aligned}$$

**Fig. 4:**
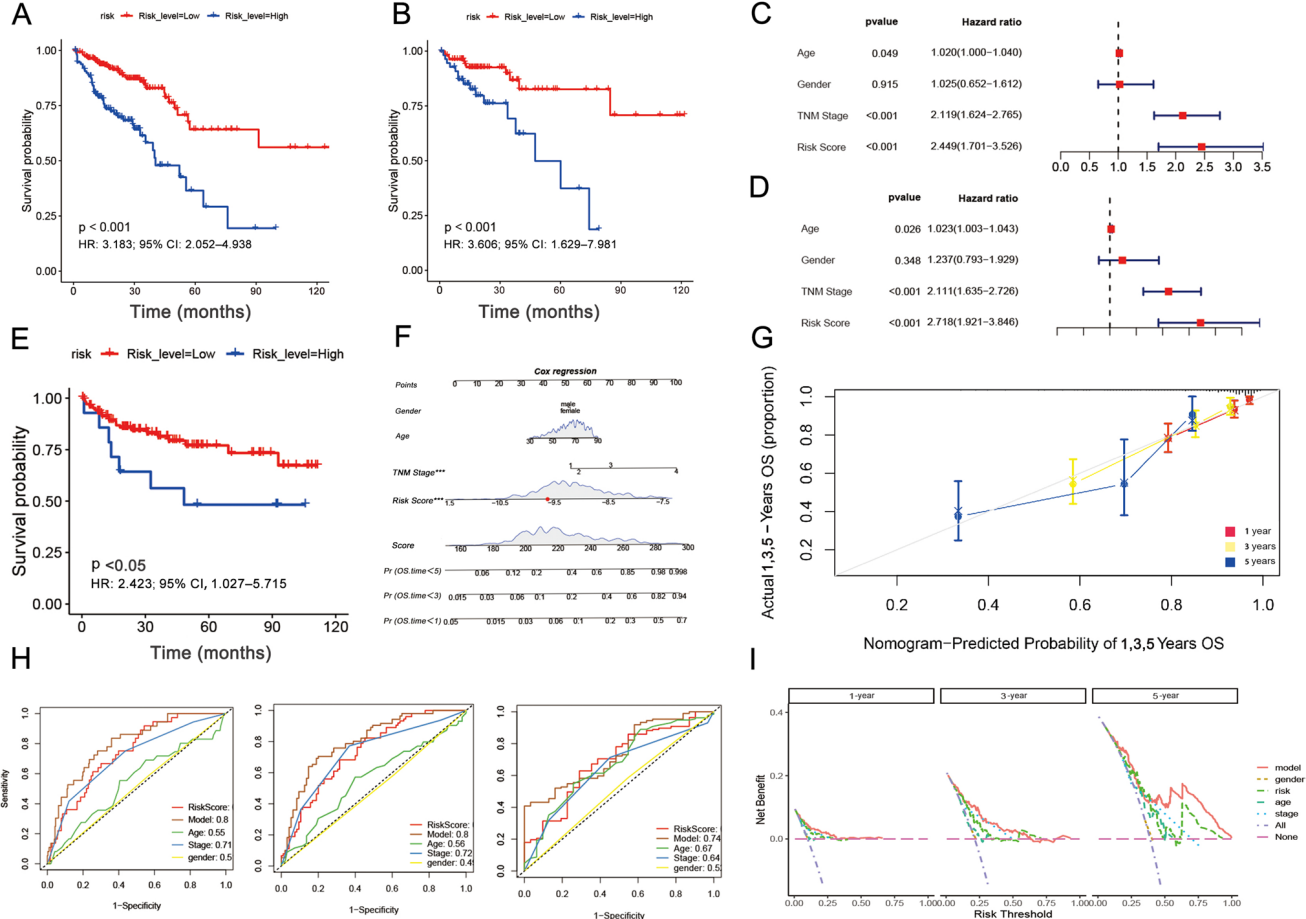
Internal and external validation of SRF models. **A** KM curves in the TCGA training set. **B** KM curves in the TCGA testing set. **C** Univariate and **D **multivariate Cox regression analyses of the risk signature and different clinical parameters. **E** KM curves in the GSE38832. **F** A nomogram combining the risk score, age, gender, and TNM stage. **G** Curve of calibration of the nomogram for estimating 1-, 3-, and 5-year OS **H** Time-dependent ROC curves of the nomogram for estimating 1-, 3-, and 5-year OS. **I** DCA curves of risk score in prognosis prediction of colorectal cancer in TCGA training set

The risk scores of individuals in the TCGA-CRC cohort were predicted with the help of the abovementioned formula. Individuals were classified into high- and low-risk categories as per the optimal threshold score. Additionally, PCA indicated significant clustering of individuals in the low- and high-risk categories in both the training and validation groups (Supplementary Figure S2). The KM curves also established that individuals in the high-risk category had a more unfavorable prognosis (HR: 3.183; 95% CI 2.052–4.938; *P* < 0.001) (Fig. 4A). As for the validation set, the low-risk category had considerably improved OS than the high-risk category based on the KM curves (HR: 3.606; 95% CI 1.629–7.981; *P* < 0.001) (Fig. 4B). Also, univariate Cox regression analysis implied that SRF was a risk factor for OS in the TCGA-CRC cohort (HR: 2.449; 95% CI 1.701–2.526; *P* < 0.001) (Fig. 4C). On the other hand, the multivariate Cox regression analysis established that SRF was an independent risk factor for OS in the TCGA-CRC cohort (HR: 2.718; 95% CI 1.921–3.846; *P* < 0.001) (Fig. 4D).

Furthermore, subgroup analysis demonstrated that patient survival was related to age, sex, N stage, M stage, and TNM stage for both risk groups (Supplementary Figure S3). SRF also exhibited predictive performance in various subgroups. The obtained results of the external validation cohorts GSE38832 were congruent with those of the TCGA-CRC cohort. Additionally, KM survival analysis showed improved prognosis in the low-risk category relative to the high-risk category (HR: 2.423; 95% CI 1.027–5.715; *P* < 0.05) (Fig. 4E). Owing to the strong correlation between risk scores and patient outcomes, a nomogram was developed, integrating risk scores and clinical parameters for estimating 1-, 3-, and 5-year OS (Fig. 4F). Moreover, the calibration curve of the nomogram exhibited a high consistency between the actual versus predicted OS rates (Fig. 4G). In addition to this, ROC curves were estimated to anticipate the 1-, 3-, and 5-year OS (Fig. 4H). The DCA (decision curve analysis) findings implied that the prognostic model displayed good net benefit for 1-, 3-, and 5-years (Fig. 4I). Notably, SRF was better than the TNM stage in estimating CRC prognosis. Overall, the predictive power of SRF was much better than that of clinical parameters.

### Association of immune checkpoints with risk scores

**Fig. 5:**
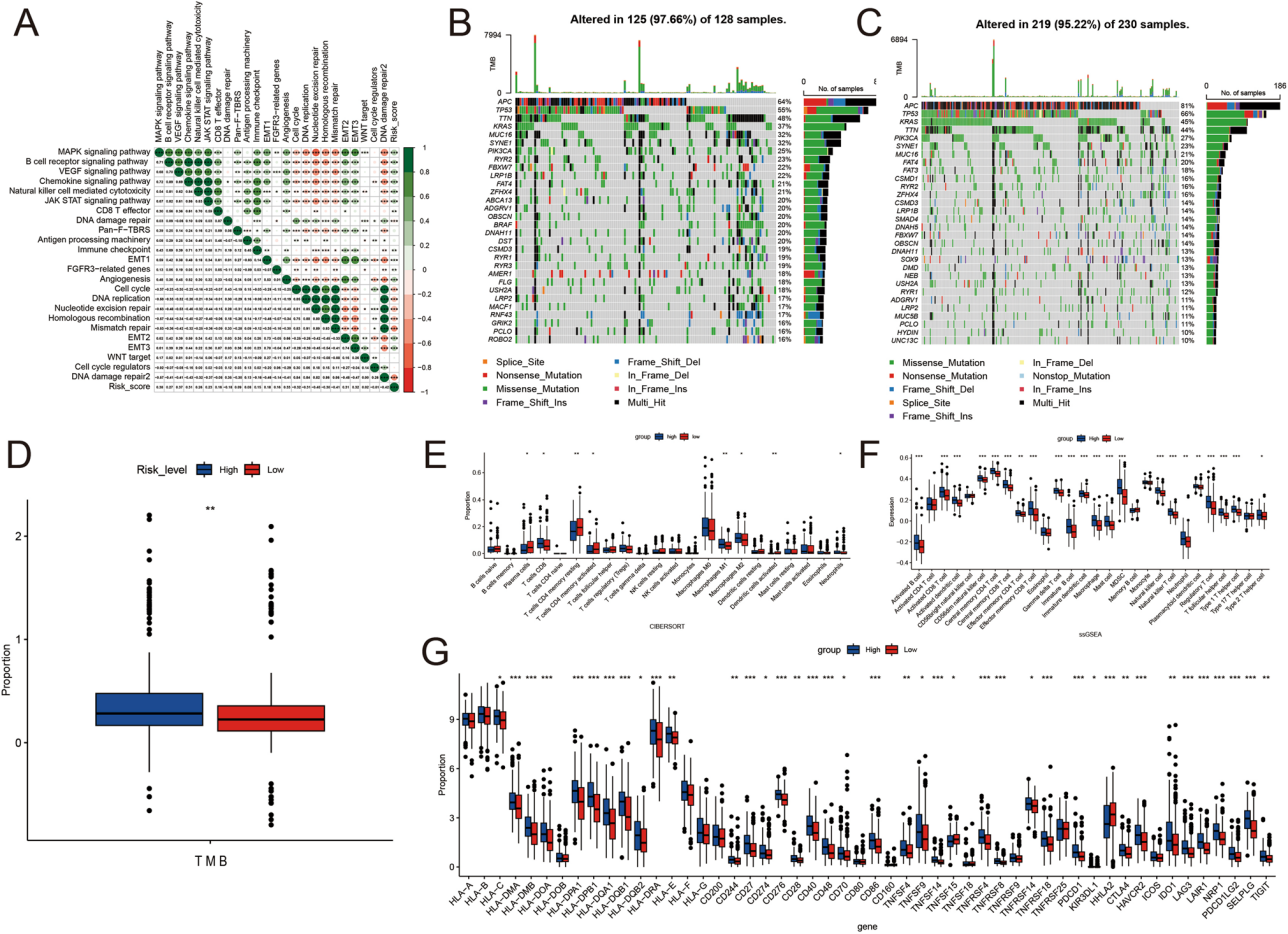
Link between the risk score and immune checkpoint expression. Mutation profile of the high-risk categories (**B**) and low-risk categories (**C**). TMB in high- versus low-risk categories (**D**). Variations in the relative abundance of infiltrating immune cells in the TME in the high- versus low-risk categories. Variations of > 0 indicate immune cell enrichment in the low-risk category, and column colors depict statistical significance. **E** Boxplots depicting the CIBERSORT scores of 22 immune cells in high- versus low-risk categories in TCGA training set. **F** Boxplots depicting the SSGSEA scores of 28 immune cells in high- versus low-risk categories in TCGA training set. **G** Expression of HLA family genes and immune checkpoints. **E** Link between the risk score and the expression of HLA family genes and immune checkpoints. TMB: tumor mutational burden; (*P < 0.05; **P < 0.01; ***P < 0.001)

Owing to the strong correlation of the risk score with immune-linked cascades, for instance, immune checkpoints, antigen-processing cells, and CD8 T effector cells (Fig. 5A), it was hypothesized that the risk score was linked to immunotherapy. Therefore, immunotherapy-relevant parameters, such as TMB and immune checkpoint expression, were investigated. Gene mutation profiles of these highly mutated genes are shown in Fig. 5B and C. TMB was elevated in the high-risk category relative to the low-risk category (*P* < 0.01) in Fig. 5D. In addition to this, the risk score was linked to the abundance of tumor-infiltrating immune cells (TIICs), including activated dendritic cells, natural killer cells, activated CD8 T cells, and activated B cells (Fig. 5E). Furthermore, variations in the cellular composition of TME between the two risk groups were also observed (Fig. 5F). The low-risk category had elevated amounts of activated dendritic cells, plasma cells, resting memory CD4 cells, and activated memory CD4 T cells. On the contrary, the high-risk category had a higher abundance of natural killer cells, M1 and M2 macrophages, activated CD8 T cells, and neutrophils, thereby demonstrating an activated immune system in high-risk individuals. Collectively, these results demonstrate that the risk score is linked to immune-associated attributes, such as TMB and TIICs. The results of the Wilcoxon test also showed substantial variations in the expression of 11 HLA family genes and 29 immune checkpoints in the low- versus high-risk groups (Fig. 5G). This suggests that the risk score is strongly linked to immune checkpoints.

### Generation of subsets of SRGs and FRGs in CRC

**Fig. 6:**
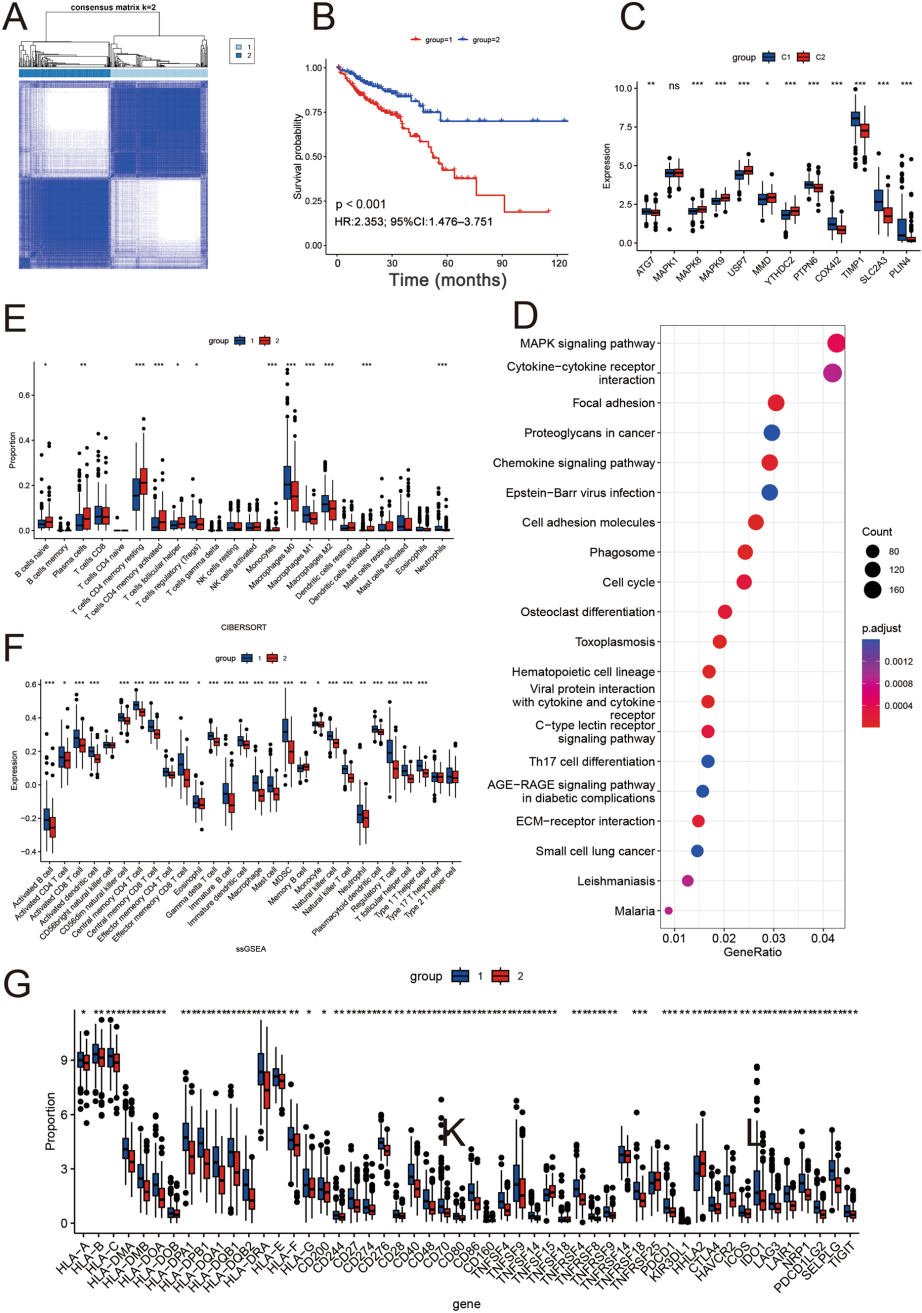
Consensus clustering analysis established molecular subtypes of CRC. **A** Consensus matrix for k value of 2. **B** Variations in OS across clusters Cluster 1 and Cluster 2. **C** Expression of disulfidptosis regulators in Cluster 1 versus Cluster 2. **D** KEGG enrichment analysis of various clusters. **E** Boxplots depicting the CIBERSORT scores of 22 immune cells in Cluster 1 versus Cluster 2 in TCGA training set. **F** Boxplots depicting the SSGSEA scores of 28 immune cells in Cluster 1 versus Cluster 2 in TCGA training set. **G** Differential expression of immune checkpoints between clusters 1 and 2 (*P < 0.05; **P < 0.01; ***P < 0.001)

To ascertain the association between the expression of SRF and the subtypes of CRC, consistency clustering analysis aided the classification of individuals with CRC as per the expression of SRF genes (Fig. 6A). The optimal clustering variable was 2. Consequently, individuals in the entire cohort were classified into clusters C1 (n = 200) and C2 (n = 188). Significant variations were observed in OS across the two clusters (HR: 2.353; 95% CI 1.476–3.751; *P* < 0.001) (Fig. 6B). The expression of genes negatively associated with prognosis was downregulated in the C1 cluster, whereas the expression of positively associated genes was elevated in the C1 cluster (Fig. 6C). Moreover, KEGG enrichment analyses of DEGs between C1 and C2 clusters established that the DEGs were enriched in the IL-17, MAPK, Ras, mismatch repair, and other cancer- and immune-related pathways (Fig. 6D).

To ascertain the link between FRGs and TME in CRC, the abundance of infiltrating immune cells in both clusters was estimated with the help of the SSGSEA and CIBERSORT algorithms. As illustrated in Fig. 6E, F, significant variations were observed in the immune cell abundance across the two clusters. The abundance of activated CD8 T cells, activated CD4 T cells, activated dendritic cells, natural killer cells, natural killer T cells, and neutrophils was elevated in the C1 cluster. Additionally, owing to the significance of immune checkpoints for tumor immunotherapy efficacy, variations were explored in immune checkpoint expression across the two clusters (Fig. 6G). Eventually, it was reported that the expression level of the immune checkpoint gene was remarkably elevated in individuals from cluster 1. Therefore, as per the aforementioned analysis, it was reported that cluster 1 is more effective and sensitive for immunotherapy.

### Risk scores predicted the benefits of chemotherapy and immunotherapy

**Fig. 7:**
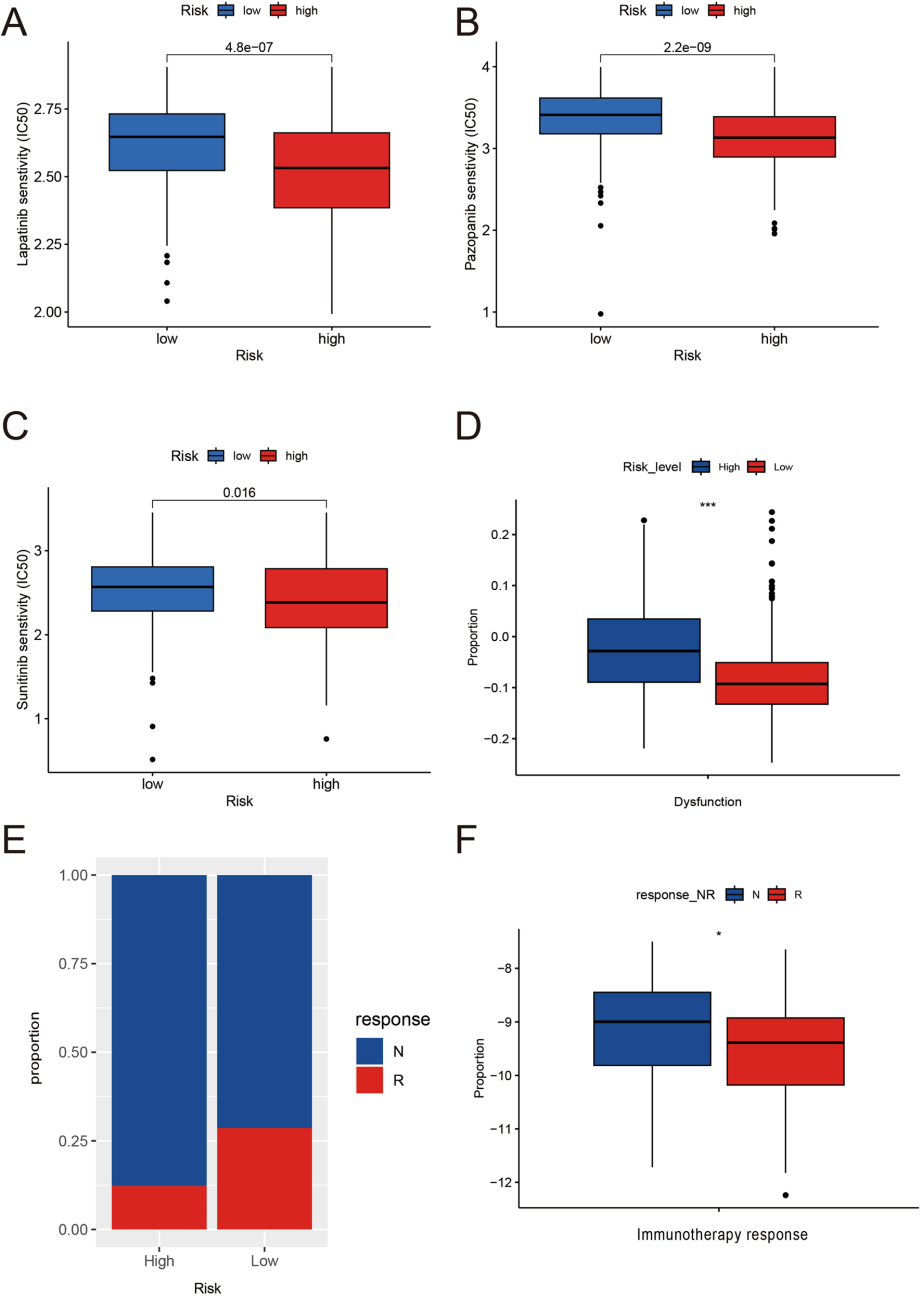
Contribution of the risk score in estimating chemotherapy and immunotherapy responses. **A**–**C** Efficacy of sunitinib, Pazopanib, Lapatinib. **D** TIDE algorithm for anticipating the response to immunotherapy in the TCGA-CRC cohort. **G** The percentage stacked histogram of immunotherapy response between high-risk and low-risk. **F** SRF Risk score of individuals sensitive to PD-1 blockade therapy in the response and nonresponse categories. IC50: half-maximal inhibitory concentration; CR: complete response; PD: progressive disease; PR: partial response; SD: stable disease

To ascertain the contribution of the risk score in estimating chemotherapy response, IC50 values were computed for 138 drugs. Individuals in the high-risk category displayed enhanced sensitivity (*P* < 0.001) to sunitinib, pazopanib, and lapatinib (Fig. 7A–C), thereby suggesting that the risk score serves to indicate drug sensitivity.

In order to examine the contribution of the risk score in estimating the reaction to immune checkpoint blockade (ICB) therapy, TIDE aided ICB therapy efficacy prediction. T-cell dysfunction scores were elevated in the high-risk category relative to the low-risk category (Fig. 7D). An immunotherapy cohort (GSE91061) was even employed for external validation [28]. The risk scores of individuals subjected to immunotherapy were computed based on the expression of SRF genes, and they were segregated into high- and low-risk groups accordingly. Individuals with low-risk scores had significant therapeutic advantages and enhanced immunosensitivity to PD-1 blockade therapy (Fig. 7E) (responders/non-responders: 28.6%/12.2%, irrespective of complete remission [CR], progressive disease [PD], and partial response [PR]). Patients unresponsive to immunotherapy had significantly higher risk scores than sensitive individuals (Fig. 7F). Collectively, these results depicted that the risk score was substantially linked to the tumor immunophenotype and, thereby, effectively predicted the response of CRC individuals to ICB therapy.

### Relationship between risk scores and miRNA–mRNA regulatory networks

**Fig. 8:**
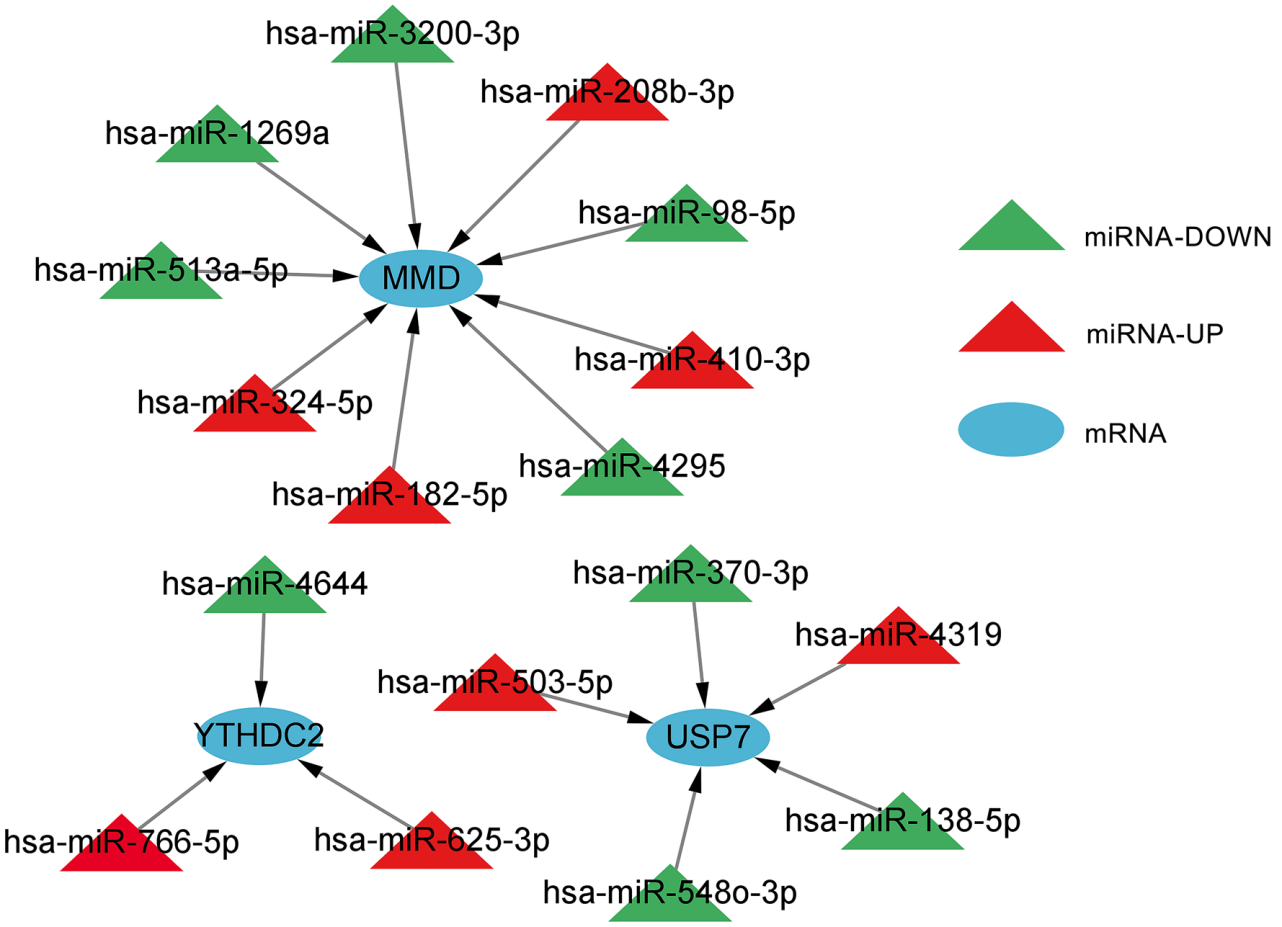
miRNA-mRNA regulatory network. The triangles depict miRNAs, whereas the circles depict mRNAs. Red represents upregulation, and green indicates downregulation. miRNA: microRNA; miR: microRNA; has: *Homo sapiens*

The risk score is a prognosis assessment tool that depends on disulfidptosis and ferroptosis regulators involved in post-transcriptional modifications. It was hypothesized that the risk score is closely linked to the expression of miRNAs in the context of Alternative polyadenylation (APA) events as a potential mechanism. TargetScan aided the prediction of hub gene-related miRNAs to obtain DEMs. Hence, 113 differentially expressed miRNA were identified in the low- vs. high-risk categories. Following the intersection of the DEMs with the differentially expressed miRNA, miRNAs lacking regulatory relationships or target genes were excluded. Three mRNAs and 17 miRNAs remained, thereby forming 17 miRNA–mRNA pairs. The relationship between these mRNAs and miRNAs is illustrated in Fig. 8.

Additionally, the diamond nodes depict miRNAs, whereas the rectangular nodes depict target genes. As for the green rectangles and red nodes, they represent downregulated and upregulated miRNAs, respectively. Next, the miRNA–mRNA network was visualized using Cytoscape. MMD was found to be regulated by several miRNAs, including hsa-miR-182-5p, -1269a, -208b-3p, -324-5p, -3200-3p, -410-3p, -513a-5p, -4295, and -98-5p. USP7 was also found to be regulated by miRNAs, such as hsa-miR-370-3p, -138-5p, -503-5p, -4319, and -548o-3p. In addition to this, YTHDC2 was found to be mediated by miRNAs like hsa-miR-4644, -625-3p, and -766-5p.

### Multidimensional validation

**Fig. 9:**
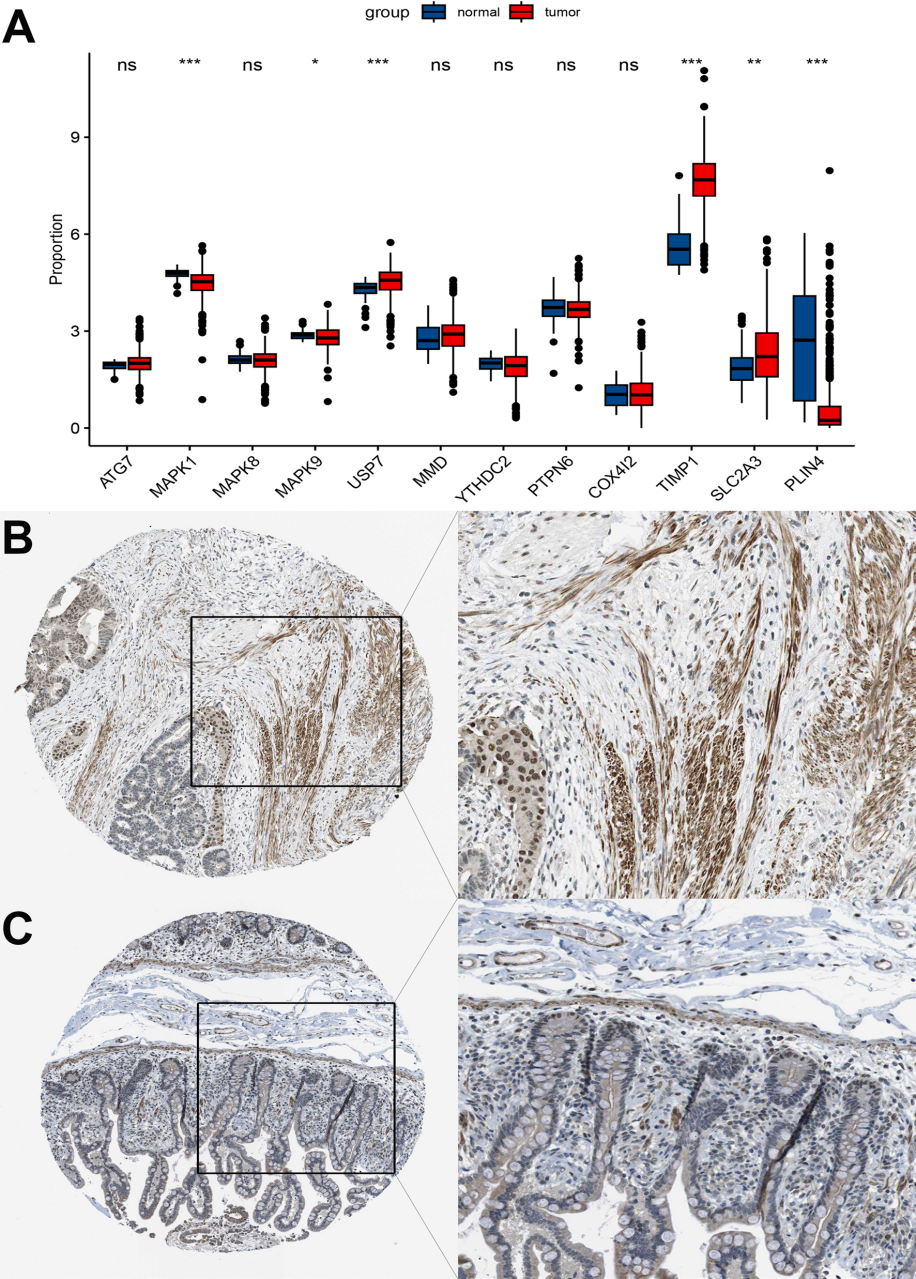
Protein-level validation of USP7. **A** Expression of Prognosis-related RFGs regulators in normal versus colorectal cancer. **B** Immunohistochemistry analysis of USP7 in normal versus colorectal tumor tissue in HPA database

USP7 was upregulated in CRC tissues (Fig. 9A). Multidimensional validation of USP7 was carried out with the help of the HPA database. USP7 stained negatively in the nuclei of CRC tissue when compared with normal tissue (Fig. 9B, C).

## Discussion

Disulfidptosis is a newly identified cell death pathway [29, 30]. Studies have highlighted the significance of disulfidptosis for the progression of bladder cancer and hepatocellular carcinoma (HCC) [29]. Ferroptosis is a potential key adaptive process for eradicating oncogenic cells. Anti-tumor strategies can exert therapeutic effects by inducing ferroptosis [14, 31]. SLC7A11, a core gene contributing to disulfidptosis, has been demonstrated to elevate oxidative stress and induce ferroptosis in CRC cells via multiple pathways [18, 32–34]. Therefore, understanding the interrelationship of disulfidptosis and ferroptosis can provide deeper insights into the antitumor immune responses, thereby guiding the development of better immunotherapeutic strategies.

Immunotherapy is a developing treatment approach for several types of tumors. Nonetheless, the advantages vary among individuals. Additionally, the link between SRGs and FRGs with the immunotherapy response, clinical characteristics, and cancer prognosis is yet to be comprehended. In the current research, the combined role of SRGs and FRGs was investigated in CRC by examining global alterations at the genetic and transcriptional levels as well as their interrelationship in CRC. The obtained findings can accelerate the development of effective immunotherapeutic treatments for CRC.

Univariate analysis of 242 SRGs and FRGs revealed 12 genes linked to CRC prognosis. Following consistent clustering analysis of these 12 genes, individuals with CRC were classified into two clusters. Most genes negatively linked to prognosis were under upregulation in the C2 cluster, and individuals exhibited an unfavorable prognosis. The USP7 and YTHDC2 genes depicted an elevated expression level in the C2 cluster. Elevated expression levels of USP7 may suppress ferroptosis, which facilitates cancer cell proliferation, thereby causing an unfavorable prognosis. Several studies have reported that YTHDC2 expression is linked to unfavorable prognosis in colorectal, breast, and gastric cancers [35–39], which is congruent with the findings of the current study. Differential expression analysis of two clusters using the limma package uncovered 10,376 common DEGs. KEGG enrichment analyses identified that these DEGs genes were under enrichment in cancer-linked pathways such as chemokine signaling, MAPK signaling, and Th17 cell differentiation-associated pathway.

SRF was developed through screening and modeling 12 prognosis-linked genes based on several machine-learning algorithms. Risk scores were computed using SRF to anticipate the prognosis of CRC individuals. High-risk individuals exhibited unfavorable OS, implying that high risk scores indicate an unfavorable prognosis.

PLIN4, as the sole gene associated with adverse prognosis, warrants further investigation. The PLIN family is widely implicated in various biological activities, including involvement in intracellular signaling, cytoskeletal organization, and regulation of lipid metabolism [40, 41], and is associated with the development and invasive metastasis of various malignant tumors. Myocytes, neurons, and others rely on PLIN4 to facilitate mitochondrial utilization of lipid droplets for β-oxidation under inflammatory and oxidative stress conditions [42]. While β-oxidation can generate more ATP compared to oxidative phosphorylation, it also generates more reactive oxygen species, consuming more oxygen and potentially damaging tissue cells. Furthermore, PLIN4 mutations have been identified in gastric cancer and lung cancer and are associated with adverse prognosis [43]. This is consistent with our findings (HR: 1.291; 95% CI 1.056–1.577; *P* < 0.05), adding credibility to our study.

A nomogram integrating risk scores and clinical parameters was developed with the purpose of anticipating 1-, 3-, and 5-year OS. The risk score was computed via genes linked to prognosis, i.e., SRGs and FRGs. However, no prior studies predicting prognosis in CRC had been reported. AUC values were estimated to authenticate the distinguishing power of the prognostic nomogram. The values ranged from 0.672 to 0.733 for 1-, 3-, and 5-year OS. The calibration curve and nomogram were congruent with the 45-degree line, thereby depicting consistency of predicted versus actual OS at 1, 3, and 5 years. Overall, the nomogram depicts a more practical tool for guiding appropriate individualized therapies for individuals with CRC, ultimately resulting in improved clinical outcomes.

The expression level of PD-L1 and CTLA-4 was remarkably elevated in the low-risk category relative to the high-risk category, congruent with literature findings [44, 45]. ICIs are common immunomodulatory monoclonal antibodies. Cytotoxic T lymphocyte-associated antigen 4 (CTLA-4) is one of the immune checkpoints that contributes as a co-inhibitory receptor on the T-cell surface. CTLA-4 is a negative regulatory molecule in T-cell stimulation, repressing the binding of CD28 to MHC and subsequently inhibiting T-cell immune responses. Programmed cell death-1 receptor binds to its ligand PD-L1 to modulate sufficient repressive signaling and prevent T effector cell proliferation. This, in turn, negatively influences anti-tumor immunity [46, 47]. Therefore, individuals with high PD-L1 and CTLA-4 expression have a poorer prognosis, as reported in the literature. Moreover, the external validation cohort (GSE91061) results strongly supported the hypothesis that the risk score is a predictor of immunotherapy response in individuals with CRC [28].

Furthermore, patients with high-risk scores were more sensitive to sunitinib, pazopanib, and lapatinib. Hence, the combination of USP7 inhibitors and chemotherapy could reduce drug resistance in individuals with chemotherapy-resistant breast cancer [48]. USP7 inhibitors sensitize cancer cells to chemotherapy by decreasing SAMHD1. This implies that the stabilization of SAMHD7 by USP7 enhances DNA damage repair to overcome oncogenic stress and influences chemotherapeutic sensitivity [49]. Additionally, the knockdown of ATG7 has been demonstrated to improve chemotherapeutic sensitivity in CRC cells [50], which is consistent with the high expression trend of USP7 and ATG7 in the low-risk category. Overall, the risk score can help ascertain drug sensitivity and clinical response to immunotherapy among individuals with CRC.

miRNA–mRNA regulatory networks were generated to study the mechanisms of genes involved in SRF. The risk score was linked to miRNA expression, which targets the 3′-UTR of genes, regulates gene expression, and also participates in cancer progression. A total of 17 miRNAs and three mRNAs formed the regulatory network, and genes mediating CRC development were also included. A thorough analysis indicated that miRNAs and mRNAs such as hsa-miR-1269a, -4295, and -4319 also contribute to CRC onset and progression. Elevated expression levels of has-miR-1269a have been linked to recurrence and metastasis in disease-free individuals with stage-II CRC. Additionally, it supports epithelial–mesenchymal transition (EMT) and facilitates CRC metastasis in vivo [51, 52]. hsa-miR-4295 is widely expressed in vivo and is linked to the progression of several tumors, including gastric cancer [53], lung cancer [54], bladder cancer [55], and squamous cell carcinoma of the head and neck [56]. As a cancer-linked miRNA, miR-4319 mediates the self-renewal ability of stem cells and tumorigenesis in multiple cancers, including CRC [57, 58], as congruent with study findings.

## Conclusion

Data from TCGA and GEO databases aided in ascertaining the association of risk scores with clinical features and immunotherapy response. A nomogram was constructed to estimate OS at 1, 3, and 5 years. The nomogram also exhibited good predictive performance and was, thereby, reported reliable for evaluating CRC prognosis.

## Limitation

The present study has certain limitations. Firstly, data collection relied on a public database for this study. Hence, additional validation utilizing diverse external datasets is necessary. Secondly, further validation of the study's findings requires in vitro and in vivo studies.

### Supplementary Information

Below is the link to the electronic supplementary material

Supplementary file1 (DOCX 611 KB)

## Data Availability

The datasets utilized and analyzed in this study are accessible through GEO (https://www.ncbi.nlm.nih.gov/geo/) and TCGA (https://portal.gdc.cancer.gov/). For additional information, please feel free to contact the corresponding authors.

## Abbreviations

CRC: Colorectal cancer
SRF: Disulfidptosis-related ferroptosis genes
FRGs: Ferroptosis-related genes
SRGs: Disulfidptosis-related genes
GEO: Gene Expression Omnibus
LASSO: Least absolute shrinkage and selection operator
TCGA: The Cancer Genome Atlas
GO: Gene Ontology
KEGG: Kyoto Encyclopedia of Genes and Genomes
TME: Tumor microenvironment
CIBERSORT: Cell-type Identification By Estimating Relative Subsets Of RNA Transcripts
GSVA: Gene set variation analysis
ssGSEA: Single-sample Gene Set Enrichment Analysis
ROC: Receiver operating characteristic
IC50: Half maximal inhibitory concentration
AUC: Area under the curve
DCA: Decision curve analysis
TIDE: Tumor immune dysfunction and exclusion
HPA: The Human Protein Atlas
