# Supplementary material for: Molecular characterization, clinical value, and cancer–immune interactions of genes related to disulfidptosis and ferroptosis in colorectal cancer
Source: Discov Oncol. 2024 May 24;15:183. doi: 10.1007/s12672-024-01031-y (PMC11126553; doi:10.1007/s12672-024-01031-y)
Supplement: Supplementary file 1 — Supplementary file1 (DOCX 611 KB) [file 12672_2024_1031_MOESM1_ESM.docx]

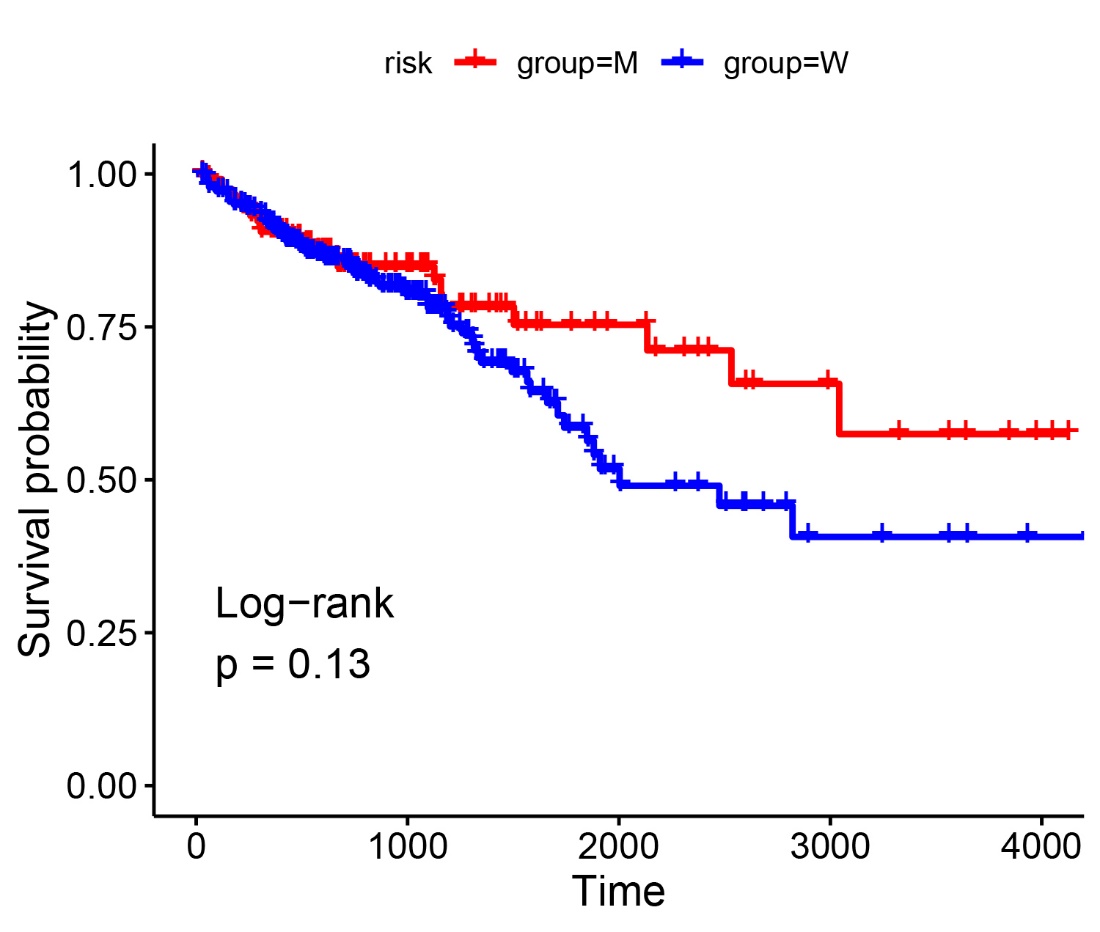


Figure S1 Kaplan-Meier curves of survival in the mutate and wild TMB groups in the TCGA-CRC cohort.


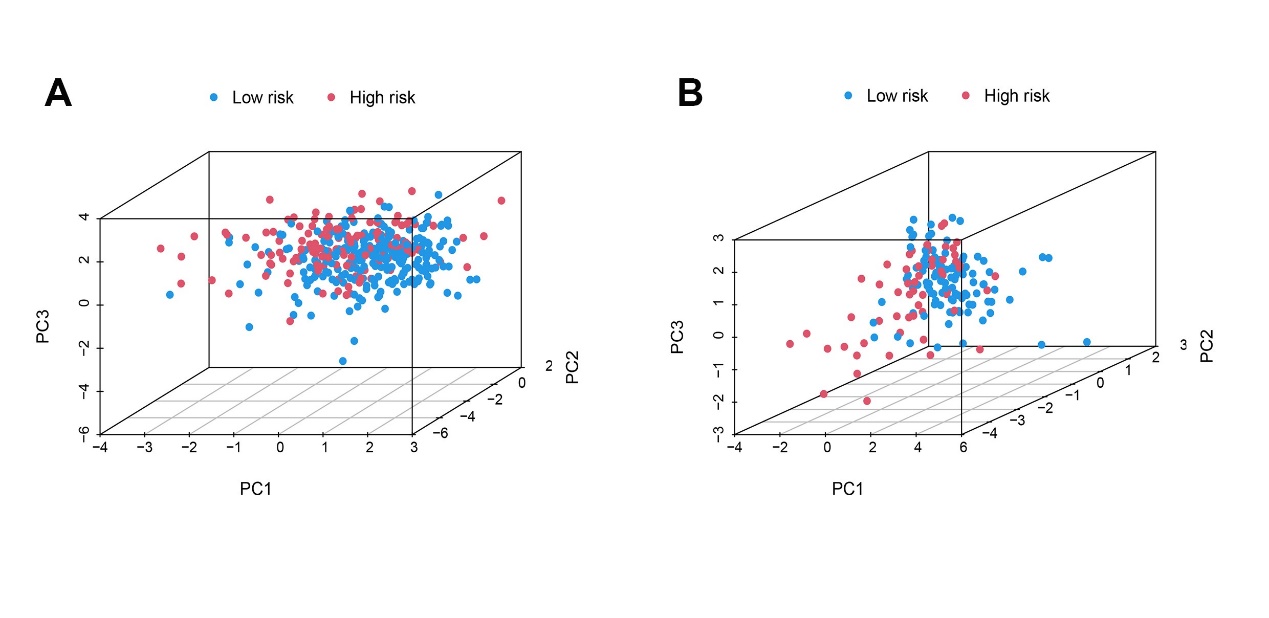


Figure S2 (A)TCGA training cohort PCA Plot. (B) TCGA testing cohort PCA Plot


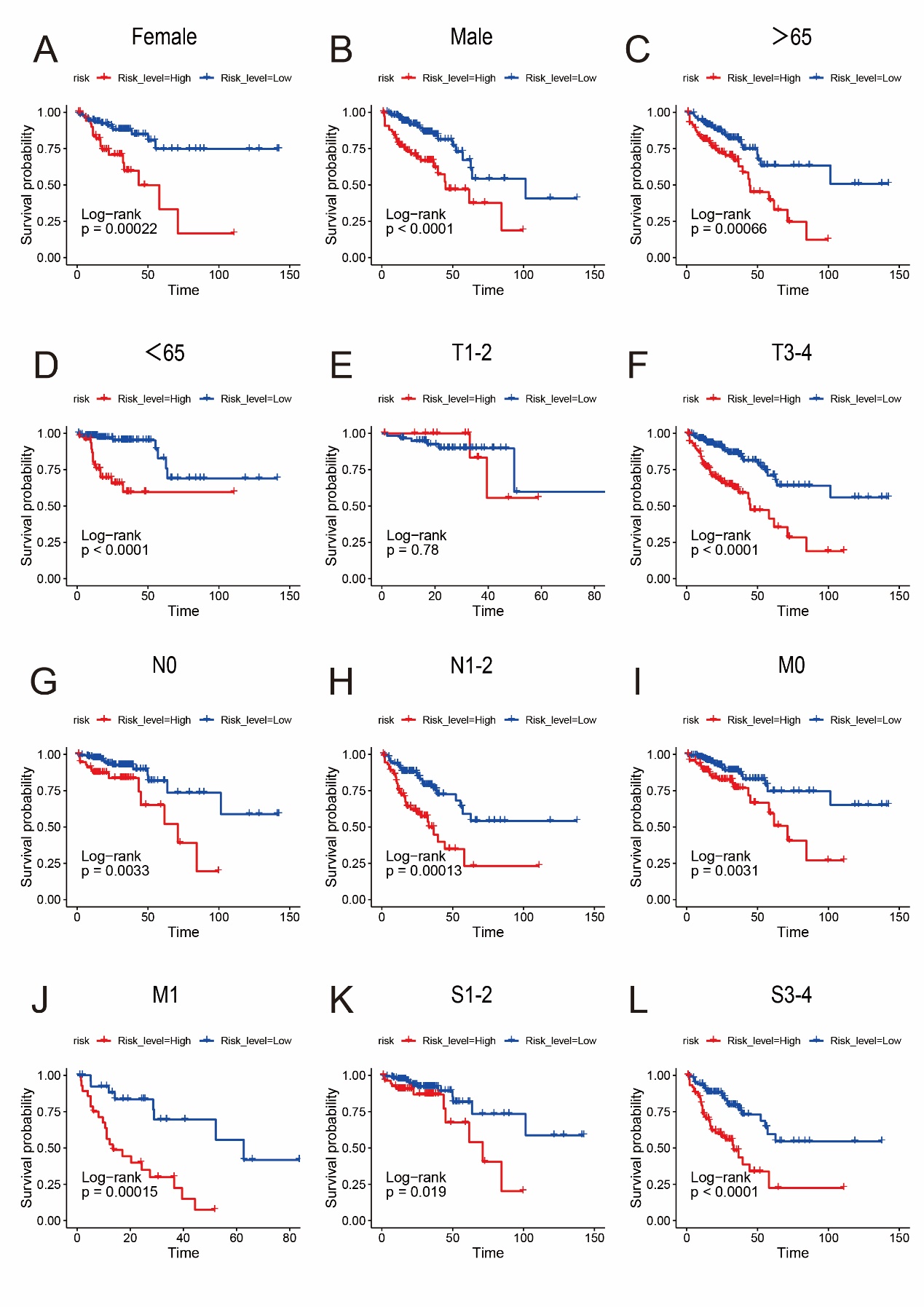


Figure S3 The risk score, as per the SRF signature, is a useful marker for an unfavorable prognosis in different subgroups classified by clinicopathological attributes. The SRF could identify high-risk individuals in different subgroups classified by clinicopathological features such as (A, B) gender, (C, D) age, (E, F) T stage, (G, H) N stage, (I, J) M stage (K, L) TNM stage.
